# BLF1 Affects ATP Hydrolysis Catalyzed by Native and Mutated eIF4A1 and eIF4A2 Proteins

**DOI:** 10.3390/toxins17050232

**Published:** 2025-05-07

**Authors:** Min An, Xin Cheng, Yu Zhang, Jiang Gu, Xuhu Mao

**Affiliations:** 1 Department of Clinical Microbiology and Immunology, College of Pharmacy and Medical Laboratory, Army Medical University (Third Military Medical University), Chongqing 400038, China; man223@tmmu.edu.cn (M.A.); zy1967394686@163.com (Y.Z.); 2 Department of Biochemistry and Molecular Biology, School of Basic Medicine, Army Medical University (Third Military Medical University), Chongqing 400038, China; 3 Department of Microbiology and Biochemical Pharmacy, College of Pharmacy and Medical Laboratory, Army Medical University (Third Military Medical University), Chongqing 400038, China; chengxin2009@tmmu.edu.cn (X.C.); jianggu2012@163.com (J.G.); 4 College of Medical Technology, Chengdu University of Traditional Chinese Medicine, Chengdu 611137, China

**Keywords:** eIF4A mutations, cancer treatment, eukaryotic initiation factor 4A, *Burkholderia pseudomallei*, recombinant proteins

## Abstract

*Burkholderia* lethal factor 1 (BLF1), a toxin derived from *Burkholderia pseudomallei*, reacts with eukaryotic initiation factor (eIF) 4A to inhibit protein synthesis. eIF4A1 and eIF4A2 are involved in translation initiation and share over 90% sequence similarity. However, they exert distinct effects on cancer treatment outcomes. To understand the molecular mechanism by which BLF1 modulates eIF4A isoforms in cancer cells, we investigated its effects on eIF4A-mediated adenosine 5′-triphosphate (ATP) hydrolysis. We found that eIF4A1 has a higher ATP-binding affinity compared to eIF4A2 (K_m_ = 6.55 ± 0.78 μM vs. K_m_ = 11.61 ± 2.33 μM). Meanwhile, we also found that eIF4A1 is more sensitive to changes in temperature, pH, and Mg^2+^ concentration. Through N-terminal swapping and single amino acid mutations, we found that leucine 98 (L98) and alanine 100 (A100) play important roles in the ATPase activities of eIF4A isoforms. Moreover, BLF1 treatment significantly enhanced eIF4A2-mediated ATP hydrolysis at all tested ATP concentrations. These differences in BLF1-regulated eIF4A isoforms may explain its selective cytotoxicity against cancer cells. Our findings provide molecular insights into the functional difference between eIF4A isoforms and suggest that BLF1 might be of promising value for anticancer therapies.

## 1. Introduction

Burkholderia lethal factor 1 (BLF1), a toxin isolated from *Burkholderia pseudomallei*, is a protein that can modulate eukaryotic initiation factor 4A (eIF4A) proteins [1,2]. eIF4A proteins belong to the DEAD-box RNA helicase superfamily and exhibit adenosine 5′-triphosphate (ATP)-dependent RNA-binding and -unwinding activities [3,4]. BLF1 can deaminize glutamine 393 in eIF4A, making it unable to unwind the secondary cap structure in the 5′-untranslated region (5′-UTR) of mRNA [1,2,5]. This inhibits ribosome screening from the start codon and halts protein synthesis, leading to cell death [1,2,5]. These characteristics of BLF1 have sparked research interest in its use for developing anticancer drugs [2,6,7,8]. Therefore, understanding the molecular mechanism of BLF1 is essential for evaluating its therapeutic potential.

Recent evidence has demonstrated that eIF4A proteins predominantly adopt an open conformation, which not only affects their ATPase activity but also influences their RNA-binding capabilities [3,4]. During ATP hydrolysis, the conformation of eIF4A proteins switches from the open state to the closed state, which is critical for RNA unwinding [4]. When assembled into the translation initiation machinery, the helicase activity of eIF4A proteins is roughly 20 times greater than that of their free form [9]. This observation suggests that proper ATP hydrolysis is important for the regulation of eIF4A function. In addition, the ATP-hydrolyzing activity of eIF4A, although not directly involved in protein translation initiation, is crucial for protein synthesis by modulating RNA granule formation [10]. Cap-dependent initiation is the most common first step in protein synthesis, wherein the eIF4A protein unwinds the secondary structure in the 5′-UTR of mRNAs in the presence of ATP, initiating ribosome screening and leading to protein synthesis [11,12,13]. Among the three eIF4A isoforms identified in human cells, eIF4A1 and eIF4A2 are involved in protein synthesis in the cytoplasm [14,15,16]. These two isoforms are over 90% identical in their primary sequence, and both are ATP-dependent RNA helicases [17]. Aside from their helicase and ATPase activities, these two proteins exhibit some distinct physiological functions [9]. Upon inhibition of eIF4A1, eIF4A2 can substitute for eIF4A1 to form the eIF4F complex to initiate translation. However, the protein synthesis governed by the resulting eIF4A2-containing complex is less than that controlled by the eIF4A1-containing complex [9].

eIF4A1 has been found to be significantly upregulated in lung, stomach, and liver tumors, and its elevated expression correlates to a poor clinical prognosis [16,18,19,20]. These findings reflect a dysregulated protein synthesis framework in cancer cells. Within this network, eIF4A plays a crucial role, making it a promising therapeutic target. Additionally, the differences between eIF4A1 and eIF4A2, which have been underestimated by previous studies, potentially provide an opportunity for developing selective therapeutic interventions that specifically target cancer cells while leaving normal cells intact.

As aberrant protein expression is a hallmark of cancer, regulating protein expression is a promising method to treat cancers [21,22,23]. Moreover, the immunogenicity and protective efficacy of the BLF1-N and BLF1-C terminal domains against full-length BLF1 have been demonstrated in various studies [8,24,25,26,27]. Our preliminary data, along with previous findings, show that BLF1 can kill tumor cells, leaving healthy cells intact [1].

However, the mechanism underlying this effect remains unclear. We hypothesized that the varying effects of BLF1 in tumor and healthy cells may be caused by its differential effects on eIF4A1 and eIF4A2. Previous investigations on the antitumor activity of BLF1 have focused on the cellular level [1,6,8,28]. To elucidate the molecular mechanism underlying BLF1’s selective cytotoxicity toward cancer cells while sparing normal cells at low concentrations, we characterized its effects on the ATPase activities of the eIF4A1 and eIF4A2 proteins using an in-vitro reconstitution system.

## 2. Results

### 2.1. eIF4A1 and eIF4A2 Catalyze ATP Hydrolysis Differently

**Figure 1:**
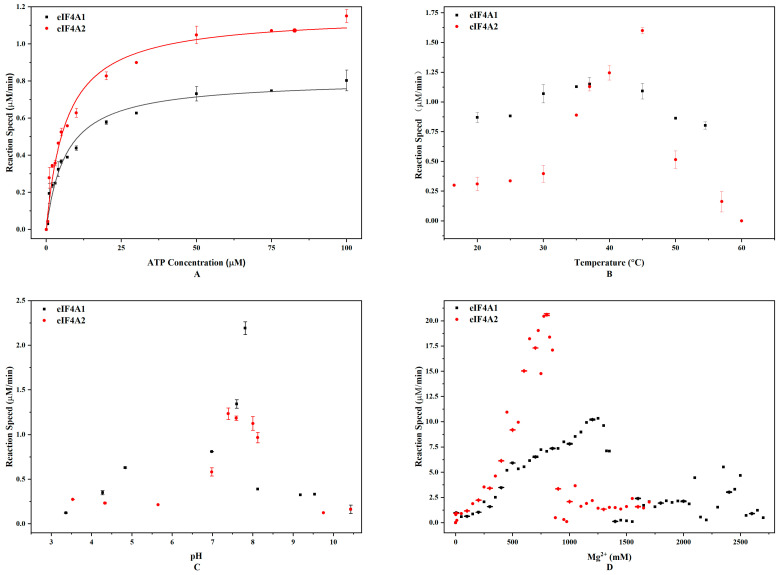
eIF4A1 and eIF4A2 catalyze ATP hydrolysis differently. (**A**) The fitting curves of ATP concentration versus reaction rate for eIF4A1 and eIF4A2. The black squares and red triangles represent the reactions catalyzed by eIF4A1 and eIF4A2, respectively. (**B**) Hydrolysis at different temperatures and in the presence of 100 μM ATP. (**C**) Hydrolysis at different pH values and in the presence of 50 μM ATP. (**D**) Hydrolysis in the presence of different Mg^2+^ concentrations and 50 μM ATP. The black squares and red circles represent the reactions catalyzed by eIF4A1 and eIF4A2, respectively.

To determine the difference between eIF4A1 and eIF4A2 in ATP hydrolysis, we evaluated their ATP hydrolysis kinetics. The results demonstrated that eIF4A1 showed a lower K_m_ (6.55 ± 0.78 μM) compared to eIF4A2 (11.61 ± 2.33 μM), indicating its higher ATP-binding affinity (Figure 1A). Meanwhile, the maximum reaction rates (V_max_) for eIF4A1 and eIF4A2 were comparable (1.16 ± 0.04 mM/min vs. 1.09 ± 0.07 mM/min, respectively).

Next, we investigated how temperature, pH, and Mg^2+^ concentration affect the ATPase activities of eIF4A1 and eIF4A2. In terms of temperature, eIF4A1 and eIF4A2 reached their V_max_ at 37 °C (1.16 μM/min) and 45 °C (1.60 μM/min), respectively (Figure 1B). In terms of pH, eIF4A1 exhibited the highest reaction rate (2.19 μM/min) at pH 7.81, whereas eIF4A2 exhibited the highest reaction rate (1.23 μM/min) at pH 7.40. Furthermore, the lowest reaction rates for eIF4A1 and eIF4A2 were approximately 0.124 μM/min and 0.125 μM/min, respectively (Figure 1C). Although eIF4A1 and eIF4A2 attained their respective V_max_ at similar pH values, the rate changes were more significant for eIF4A1. These observations suggest that eIF4A1 has a higher pH sensitivity. In addition, we also noticed that eIF4A1 and eIF4A2 reached their V_max_ at 1.3 M Mg^2+^ (7.21 μM/min) and 0.725 M Mg^2+^ (5.07 μM/min), respectively (Figure 1D). This indicates that these two proteins have different Mg^2+^ requirements.

### 2.2. BLF1 Affects eIF4A1- and eIF4A2-Catalyzed ATP Hydrolysis in Different Ways

**Figure 2:**
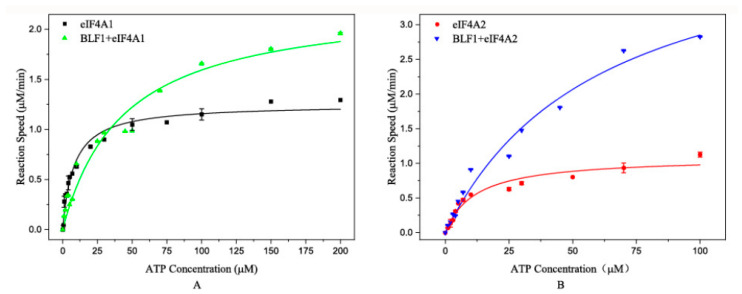
Effects of BLF1 on eIF4A1- and eIF4A2-catalyzed ATP hydrolysis. (**A**) Effects of BLF1 on eIF4A1-catalyzed ATP hydrolysis. The black squares and green triangles represent the reactions catalyzed by eIF4A1 and BLF1-treated eIF4A1, respectively. (**B**) Effects of BLF1 on eIF4A2-catalyzed ATP hydrolysis. The red circles and blue triangles represent the reactions catalyzed by eIF4A2 and BLF1-treated eIF4A2, respectively. The curves in the figures were generated by fitting experimental data to a Michaelis-Menten equation.

To investigate whether BLF1 affects eIF4A activity, we determined the ATPase activities of eIF4A1 and eIF4A2 with or without BLF1. The results demonstrated different effects of BLF1 on the ATP hydrolysis patterns of eIF4A1 and eIF4A2 (Figure 2). Specifically, BLF1 incubation moderately elevated the V_max_ of eIF4A1 (from 1.3 μM/min to 2 μM/min) and increased the ATP saturation concentration (from 10 µM to approximately 100 μM) (Figure 2A). In contrast, BLF1 enhanced the eIF4A2 reaction rates independently of the ATP concentration (Figure 2B).

Consistently, kinetic analyses validated these differences. We observed that BLF1 incubation increased the K_m_ of eIF4A1 from 6.55 ± 0.78 μM to 43.58 ± 8.58 μM. We also noticed that BLF1 treatment increased the K_m_ of eIF4A2 from 11.61 ± 2.33 μM to 61.18 ± 12.66 μM. Moreover, BLF1 treatment doubled the V_max_ of eIF4A1 (from 1.16 ± 0.04 to 2.29 ± 1.72 μM/min) and boosted that of eIF4A2 by approximately fourfold (from 1.09 ± 0.07 to 4.60 ± 0.50 μM/min).

### 2.3. ATP Hydrolysis Catalyzed by the Mutated Proteins

To further dissect the difference in enzymatic changes in ATP hydrolysis between eIF4A1 and eIF4A2, protein sequence alignment was performed. The results showed that the major differences were at the N-terminal region (Supplementary Figure S5), which contains the catalytic center for ATP hydrolysis [13]. Based on these results, we generated two types of eIF4A protein mutations. The first type was an N-terminal region swap mutation. Specifically, the N-terminal regions of eIF4A1 and eIF4A2 were exchanged, resulting in eIF4A1 Mut (containing the N-terminus of eIF4A2) and eIF4A2 Mut (containing the N-terminus of eIF4A1). The other mutation type was a single amino acid mutation in eIF4A1, which replaced specific amino acids with the corresponding ones from eIF4A2 (I94L, L96I, D97E, L98F, and A100E).

**Figure 3:**
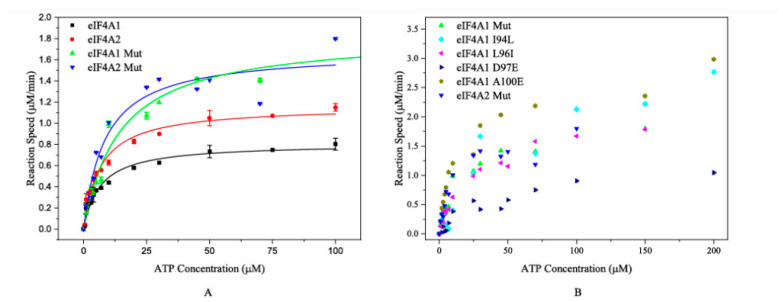
The plots of the reaction rates of mutated proteins versus ATP concentrations. (**A**) The fitting curves for eIF4A1, eIF4A1 Mut, eIF4A2, and eIF4A2 Mut. The black squares, red circles, green triangles, and blue triangles represent the reactions catalyzed by eIF4A1, eIF4A2, eIF4A1 Mut, and eIF4A2 Mut, respectively. (**B**) ATP hydrolysis catalyzed by other mutated proteins. The reactions catalyzed by eIF4A1 Mut and eIF4A2 Mut are also shown. The green and blue triangles represent the reactions catalyzed by eIF4A1 Mut and eIF4A2 Mut, respectively.

Subsequently, the ATP-hydrolyzing activities were determined using wild-type and/or mutated proteins. The results demonstrated that eIF4A1 Mut exhibited a comparable K_m_ to eIF4A2 (15.71 ± 1.51 μM vs. 11.61 ± 2.33 μM). Meanwhile, the K_m_ of eIF4A2 Mut and eIF4A1 were also similar (7.85 ± 0.54 μM and 6.56 ± 0.78 μM, respectively) (Figure 3A and Table 1). These results suggest that the N-terminal sequence plays critical roles in the ATP-binding and ATP-hydrolyzing activities of these proteins. In parallel, we investigated the ATP-hydrolyzing capability of the single-mutated proteins. The K_m_ values of eIF4A1 I94L (35.35 ± 2.10 μM), L96I (26.65 ± 1.41 μM), and D97E (36.89 ± 2.05 μM) were significantly greater than both wild-type eIF4A1 (6.56 ± 0.78 μM) and wild-type eIF4A2 (11.61 ± 2.33 μM) (Figure 3B and Table 1), indicating that these single mutations affected the ATP-binding affinity. In contrast, the eIF4A1 A100E mutation had a K_m_ value of 10.55 ± 0.25 μM, which is comparable to that of eIF4A2 (11.61 ± 2.33 μM) while the eIF4A1 L98F mutation abolished its ATP-hydrolyzing activity. Collectively, these findings reveal the key amino acid residues in the N-terminal region driving the different ATPase activities of eIF4A1 and eIF4A2. More importantly, the 98th and 100th residues play important roles in the catalytic activity of eIF4A2.

### 2.4. BLF1 Also Affects Mutated eIF4A-Catalyzed ATP Hydrolysis

**Figure 4:**
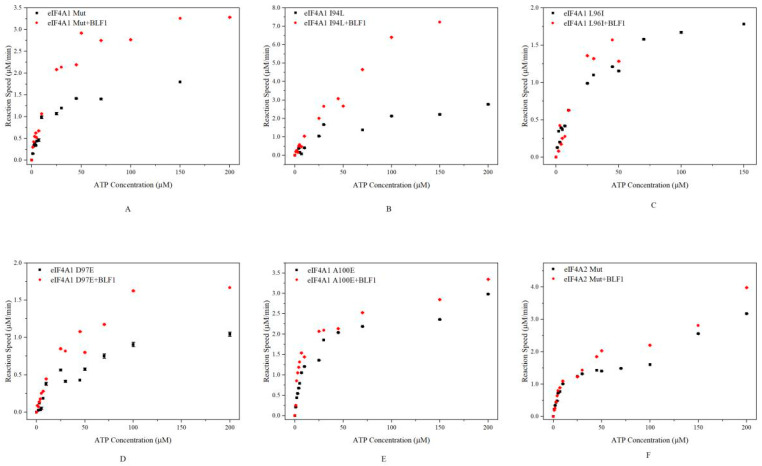
Effects of BLF1 on ATP hydrolysis catalyzed by eIF4A1 Mut (**A**), eIF4A2 Mut (**B**), eIF4A1 I94L (**C**), L96I (**D**), D97E (**E**), and A100E (**F**). As eIF4A1 L98F lost the ability to catalyze ATP hydrolysis, we have not included the relevant data here. The black triangles and red rhombi represent the reactions catalyzed by the mutated proteins and BLF1-treated mutated proteins, respectively.

We further investigated whether BLF1 affects ATP hydrolysis catalyzed by mutated eIF4A proteins. Both eIF4A2 Mut and eIF4A1 Mut responded to BLF1 when the ATP concentration was greater than 10 μM (Figure 4A,F). Furthermore, although leucine and isoleucine have similar properties, BLF1 exhibited distinct effects on eIF4A1 L96I and eIF4A1 I94L (Figure 4B,C). BLF1 enhanced the reaction rates of eIF4A1 I94L activity at all tested ATP concentrations, whereas its promotive effects on eIF4A1 L96I were only observed at ATP concentrations greater than 10 μM. For the mutants eIF4A1 D97E and eIF4A1 A100E, BLF1 enhanced their ATPase activities across all tested ATP concentrations (Figure 4D,E). These results suggest that BLF1 affects ATP hydrolysis catalyzed by these mutated proteins in different ways and accelerates their reaction at high ATP concentrations.

## 3. Discussion

In this study, we showed that full-length eIF4A1 and eIF4A2, sharing exactly 95.3% similarity in amino acid sequences (see Supplementary Figure S5), exhibit different enzymatic properties, which may result in their distinct roles in cancer development. The results of the effects of BLF1 on eIF4A isoforms demonstrated the differences in their regulation. We observed that BLF1 moderately promoted eIF4A1-mediated ATP hydrolysis at higher ATP concentrations and significantly enhanced the enzymatic activity of eIF4A2 under all tested ATP concentrations. A previous study revealed that BLF1 does not affect eIF4A-mediated ATP hydrolysis [4]. The difference may result from the methods used in the assays. In our study, we focused on RNA-independent ATP hydrolysis, whereas the previous study included RNA in the investigation [4]. Thus, our data revealed a previously unrecognized aspect of the regulatory mechanism by which BLF1 regulates eIF4A-mediated ATP hydrolysis. Moreover, the different responses of eIF4A isoforms to BLF1 may be attributed to the structural characteristics of eIF4A1 and eIF4A2. It has been demonstrated that eIF4A2 adopts an “open” conformation while it catalyzes ATP hydrolysis, which affects its RNA-binding capacity [15,16]. Thus, we speculated that BLF1 increases ATP hydrolysis while concomitantly regulating RNA binding, which contributes to its different regulatory effects on eIF4A isoforms. Although previous investigations assumed that eIF4A1 and eIF4A2 are functionally comparable because of their high animo acid sequence similarity, our results indicated that these two proteins are mechanistically distinct. However, there are not enough data that suggest that the ATPase activities of eIF4A1 and eIF4A2 are different inside the cells. Some previous studies suggest that these two proteins behave differently in tumor cells [14,18,29]. A reasonable inference is that the varying behaviors are caused by the distinct enzymatic activities of the two proteins, and the ATPases of these two proteins are different, as we demonstrated in in-vitro conditions.

Meanwhile, the results from the swap mutation and single amino acid mutation analyses revealed that L98 and A100 are critical for the biochemical properties of these proteins. The deletion of ATP hydrolysis capacity by the L98F mutation demonstrates that this residue is important for the enzymatic function of these proteins. Although the ability of eIF4A isotypes to catalyze ATP hydrolysis does not alter protein synthesis initiation, it does affect overall protein synthesis by influencing RNA granule formation [30,31]. As tumor cells are characterized by abnormal protein synthesis, eIF4A proteins have become popular targets in cancer research. Cytotoxic proteins from plants and microbes are promising candidate drugs for cancer treatment [24,25,26,27,32,33], and several natural proteins have been found to kill tumor cells. Previous studies have shown that BLF1 can kill tumor cells at low concentrations without affecting healthy cells [1,6]. Our data offered a potential explanation that BLF1 may adopt different regulatory mechanisms on eIF4A isoforms.

However, our study has several limitations. First, our data were generated from purified proteins, which may not fully reflect the complex intracellular and intercellular environment. In cellular contexts, the interactions between the BLF1 and eIF4A isoforms may be subjected to other regulatory mechanisms. Besides, the post-translational modifications of the eIF4A isoforms could also alter their susceptibility to BLF1 regulation. Additional investigations should be performed to validate our findings in the cellular system and in preclinical models. Second, although critical amino acid residues were identified in our study, the precise molecular mechanism remains elusive. Crystal structural analysis, including the complex involving BLF1 and eIF4A isoforms, would provide valuable information to address this enigma. Additionally, further research on the roles of eIF4A1 and eIF4A2 in cancer development is warranted.

## 4. Conclusions

Our results demonstrate that BLF1 differentially modulates eIF4A1- and eIF4A2-dependent ATP hydrolysis, with a more pronounced effect on eIF4A2. This functional selectivity provides a mechanistic explanation for the distinct responses of tumor and healthy cells to BLF1, supporting its potential as a targeted anticancer agent. These isoform-specific effects further underscore the importance of eIF4A helicases in context-dependent cellular drug responses. Together, these findings advance our understanding of BLF1 activity at the molecular level and highlight the translational value of BLF1 as an anticancer target.

## 5. Materials and Methods

### 5.1. Materials

The materials used to express and purify the proteins as well as the respective suppliers are listed in the Supplementary Materials and Methods. The Malachite Green Phosphate Assay Kit was purchased from Sigma-Aldrich (Darmstadt, Germany). 4-(2-Hydroxyethyl)-1-piperazineethanesulfonic acid (HEPES), dithiothreitol (DTT), and trisaminomethane (Tris) were purchased from Sangon Biotech (Shanghai, China). NaCl, magnesium acetate, acetic acid, sodium acetate, citric acid, sodium citrate, potassium hydroxide (KOH), sodium carbonate (Na_2_CO_3_), potassium chloride (KCl), and sodium bicarbonate (NaHCO_3_) were purchased from J&K Scientific (Beijing, China). All chemicals were used without any further purification.

### 5.2. Evaluating the Environmental Factors (Temperature, Mg^2+^ Concentration, and pH) on eIF4A1- and eIF4A2-Mediated ATP Hydrolysis

#### 5.2.1. ATP Hydrolysis Measurement

ATP hydrolysis was conducted in a reaction buffer containing 15 mM HEPES (pH 7.5), 80 mM KCl, 2.5 mM magnesium acetate, and 1 mM DTT. ATP stock solution (5 M) was prepared in reaction buffer and stored at 4 °C. During the experiments, the stock solution was diluted with reaction buffer to the required concentrations. For each experiment, 2 μM eIF4A1 or eIF4A2 protein was added to reaction buffer containing the desired amount of ATP to a total volume of 80 mL and incubated at 37 °C for 25 min, unless indicated otherwise. The reaction was stopped by cooling at 4 °C for 5 min, followed by mixing with 160 mL of reaction buffer. Free phosphate was measured using a Malachite Green Phosphate Assay Kit, according to the manufacturer’s instructions. Subsequently, 90 mL of the reaction mixture was transferred to 96-well plates in triplicate, and the absorbance was determined at 620 nm. The free phosphate concentration was determined using a standard curve generated with H_3_PO_4_ (1 M) (Supplementary Figure S1). ATP hydrolysis was calculated from the difference in phosphate concentrations between reactions with and without the eIF4A1/eIF4A2 proteins, and ATP autohydrolysis was normalized to protein-free blanks.

To determine the effects of temperature on eIF4A1- and eIF4A2-mediated ATP hydrolysis, reactions containing 100 μM ATP were performed at different temperatures (20, 25, 30, 35, 40, 45, 50, and 60 °C). To investigate how pH affects ATP hydrolysis catalyzed by eIF4A1 and eIF4A2, reactions containing 50 μM ATP were performed in the following buffers with the indicated pH values: acetate/citrate buffer (pH 3.5–5.5), HEPES (pH 6.0–7.5), Tris-HCl (pH 7.8–8.6), and sodium carbonate (pH 8.8–10.0). Each buffer contained 80 mM KCl, 2.5 mM magnesium acetate, and 1 mM DTT. For the Mg^2+^-associated studies, reactions containing 50 μM ATP were conducted in reaction buffer containing MgCl_2_ at the desired concentrations. All reactions were performed at 37 °C for 25 min, unless indicated otherwise. Free phosphate was determined as described above.

The eIF4A1 and eIF4A2 proteins that were added were recombinant proteins, which were expressed and purified via the methods described in the Supplemental Material (Supplementary Figures S2 and S3).

#### 5.2.2. Effects of BLF1 on Wildtype and Mutated eIF4A Proteins

To examine whether BLF1 affects the ATPase activities of eIF4A1 or eIF4A2, the amino acid sequences of these two proteins were retrieved from the National Center for Biotechnology Information and compared using DNAMAN software (version 9.0, http://www.lynnon.com/ (accessed on 10 December 2021)). We first downloaded the amino sequences from the NCBI website (Gene ID: eIF4A1:1973, eIF4A2: 1974). From the Sequence menu, the Multiple Alignment command was selected. FASTA-formatted amino acid sequences of human eIF4A1and eIF4A2 were imported consecutively through the file upload interface. The “Protein” option was activated in the alignment configuration panel to enable amino acid residue comparison. The resulting alignment output was exported in CLUSTAL format for subsequent analysis, preserving the native scoring matrix and gap parameters, as implemented in the software’s default alignment algorithm. Based on the sequence alignment results, two types of mutations in eIF4A1 and eIF4A2 were engineered. In the first type of mutation, the N-terminal regions of eIF4A1 and eIF4A2 were exchanged, resulting in eIF4A1 Mut (containing the N-terminal region of eIF4A2) and eIF4A2 Mut (containing the N-terminal region of eIF4A1). The other type of mutation involved the replacement of single amino acids in eIF4A1 with the corresponding ones from eIF4A2, including I94L, L96I, D97E, L98F, and A100E.

To determine ATP hydrolysis, 2 mM BLF1 was preincubated with wild-type or mutant eIF4A1 or eIF4A2 at 37 °C for 5 min before measurement. Negative controls were carried out without any proteins or with eIF4A alone. The reaction rates of ATP hydrolysis were plotted against various ATP concentrations at 37 °C for 25 min. The BLF1, wild-type eIF4A, and mutant eIF4A proteins were expressed and purified as described in the Supplemental Materials (Supplementary Figures S4–S6).

### 5.3. Statistical Analysis

All enzymatic data were analyzed using Origin 2019 software. The reaction rates used for the fitting curves in this study were the average reaction rates for at least three independent experiments. Thereafter, these reaction rates at different ATP concentrations were fitted into the Michaelis–Menten curve using Origin 2019. First, the data needed for the analysis were inputted; thereafter, “Analysis” was selected from the menu. The data were analyzed using the “Non-linear Fitting” command; thereafter, the “Enzymatic Kinetics” and “Michaelis–Menten” equations were selected to draw the fitting curves. The fitting curves presented in this paper all have adjusted R^2^ values greater than 0.96, suggesting that all the curves exhibit relationships between the reaction rates and ATP concentrations, in compliance with the Michaelis–Menten rules.

## Figures and Tables

**Table 1:** K_m_ values of proteins catalyzing ATP hydrolysis.

Protein	K_m_ (μM)
eIF4A1	6.55 ± 0.78
eIF4A1 Mut	15.71 ± 1.51
eIF4A1 I94L	35.35 ± 2.10
eIF4A1 L96I	26.65 ± 1.41
eIF4A1 D97E	36.89 ± 2.05
* eIF4A1 L98F	
eIF4A1 A100E	10.55 ± 0.25
eIF4A2 Mut	7.85 ± 0.54
eIF4A2	11.61 ± 2.33

* eIF4A1 L98F was enzymatically inactive; therefore, the data are not included.

## Data Availability

The original contributions presented in this study are included in the article/Supplementary Material. Further inquiries can be directed to the corresponding author.

## Supplementary Materials

The following supporting information can be downloaded at: https://www.mdpi.com/article/10.3390/toxins17050232/s1, Supplementary Figure S1: The standard curve used to calculate the concentration of free phosphate. The absorbance at 620 nm of standard phosphate solutions (0, 4, 8, 12, 16, 24, 32, and 40 μM) were recorded and subjected to linear regression. Supplementary Figure S2: BLF1 protein purification. M, standard protein marker; 1, supernatant of induced E. coli BL21(DE3) cells; 2, beads incubated with the supernatant; 3 and 4, PreScission Protease (PSP)-treated bead flow-out; 5, PSP-treated beads. The three images represent three independent experiments. Supplementary Figure S3: eIF4A1 purification. M, standard protein marker; 1, supernatant of induced E. coli BL21(DE3) cells expressing eIF4A1; 2, beads incubated with cell supernatant; 3, Reduced glutathione (GSH)-treated bead flow-out. The three images represent three independent experiments. Supplementary Figure S4: eIF4A2 purification. M, standard protein marker; 1, supernatant of induced E. coli BL21(DE3) cells expressing eIF4A2; 2, beads incubated with cell supernatant; 3, GSH-treated bead flow-out. The three images represent three independent experiments. Supplementary Figure S5: Blast results of eIF4A1 and eIF4A2 protein sequences. Supplementary Figure S6: Purified mutated proteins. 1, eIF4A1 Mut; 2, eIF4A2 Mut; 3, eIF4A1 I94L; 4, eIF4A1 L96I; 5, eIF4A1 D97E; 6, eIF4A1 L98F; 7, eIF4A1 A100E. The three images represent three independent experiments. Supplementary Table S1: Primers used in this study Supplementary Materials and Methods.
