# Supplementary material for: BLF1 Affects ATP Hydrolysis Catalyzed by Native and Mutated eIF4A1 and eIF4A2 Proteins
Source: Toxins (Basel). 2025 May 7;17(5):232. doi: 10.3390/toxins17050232 (PMC12115832; doi:10.3390/toxins17050232)
Supplement: Supplementary file 1 [file toxins-17-00232-s001.zip › toxins-3542694-supplementary.pdf]

BLF1 Affects ATP Hydrolysis Catalyzed by Native and Mutated eIF4A1 and eIF4A2 Proteins

Supplementary Material

Supplementary Materials and Methods

Materials

The *Escherichia coli* BL21(DE3) and DH5α competent cells were purchased from Promega Corporation (Madison, WI, USA). Yeast extract, tryptone, isopropyl β-D-thiogalactoside (IPTG), and agar were purchased from Sigma-Aldrich (Darmstadt, Germany). Ampicillin, NaCl, and glycerol were obtained from Sangon Biotech (Shanghai, China). The TIAN prep Mini Plasmid Kit II was purchased from Tiangen Biotech (Beijing, China). Glutathione–Sepharose 4 Fast Flow beads were purchased from GE Healthcare (Chicago, IL, USA). The reductive glutathione (GSH) was obtained from J&K Company. The Micro BCA™ Protein Assay Kit was purchased from Sangon Biotech, and SDS-PAGE Gel Quick Preparation Kit was purchased from Beyotime Biotechnology. All reagents were used without further purification. The pGEX-6p-1-BLF1 plasmid to express recombinant BLF1 protein was constructed by Chunyan Ren, a former graduate student in our laboratory. The cDNA sequences of eIF4A1 and eIF4A2 proteins were synthesized and ligated into a T4 vector by Sangon Biotech.

Expression and purification of BLF1 protein

The pGEX-6p-BLF1 plasmids were transformed into *E. coli* BL21(DE3) competent cells. Colonies harboring the plasmids were collected the next day, inoculated in Luria-Bertani (LB) broth containing 100 mg/mL ampicillin, and incubated at 37 ℃ with

agitation for more than 8 h until the optical density (OD) value reached 0.6–0.8. Protein expression was induced using 0.1 mM IPTG for 5 h at 37 ℃. Subsequently, the bacterial pellet was collected by centrifugating the culture at 10,000 rpm and 4 ℃ for 5 min. Cells were lysed with sonication in an ice water bath (power: 120 W; 30 min, with a 6 s break every 5 s). The supernatant was separated by centrifugation at 12,000 rpm and 4 °C for 20 min. Glutathione–Sepharose 4 Fast Flow beads were incubated with supernatant at 4 °C overnight and washed three times with 10 mL purification buffer (10 mM HEPES, pH 7.5, 50 mM NaCl) the next morning. The purified proteins were digested using PreScission Protease (PSP), BLF1 protein was concentrated using centrifugation in the Millipore Amcon Ultra tube at 4 °C and 4,500 rpm, its concentration was determined using the Micro BCA Protein Assay Kit, and the purity was confirmed using PAGE. The molecular weight of BLF1 was approximately 49 kDa (**Supplementary Figure S2**).

Expression and purification of eIF4A1 and eIF4A2 proteins

DNA fragments encoding eIF4A1 or eIF4A2 proteins were amplified from the synthesized T-vectors using the primers listed in Supplementary Table S1. The expression vectors were constructed by inserting the eIF4A1 or eIF4A2 encoding genes into pEGX-6p-1 plasmids and validated by sequencing. Bacterial cells harboring plasmids were cultured as described above. Next, 20 mL bacterial culture was inoculated in 2 L LB broth containing 100 mg/mL ampicillin and grown at 37 °C with agitation until the OD reached approximately 0.6–0.8. Protein expression was induced using 0.1 mM IPTG at 16 °C overnight. The bacterial cells were lysed, the supernatant was incubated with beads, and the beads were washed as described above. The recombinant proteins were eluted with 10 mM GSH. The protein was concentrated

using Millipore Amcon Ultra tube centrifugation at 4 °C, and the protein concentration was determined using the Micro BCA Protein Assay Kit. The purity of the protein was confirmed using PAGE. The molecular weight of recombinant eIF4A1 (**Supplementary Figure S3**) and eIF4A2 (**Supplementary Figure S4)** proteins was approximately 70 kDa.

Construction of expression vectors for mutated eIF4A1 and eIF4A2 proteins

As determined using Blast (**Supplementary Figure S5)**, the differences in amino acid sequences between eIF4A1 and eIF4A2 are primarily located at their N-terminals, implying that the differential ability of these two proteins to catalyze ATP hydrolysis is due to different amino acid sequences at the N-terminals. We prepared a series of expression vectors for producing mutant proteins and examined which amino acid change causes behavioral differences between eIF4A1 and eIF4A2 when catalyzing ATP hydrolysis. First, we constructed two expression vectors: eIF4A1 Mut, which expressed the N-terminus of eIF4A2 and C-terminus of eIF4A1, and eIF4A2 Mut, which expressed the N-terminus of eIF4A1 and C-terminus of eIF4A2 (the used primers are listed in Supplementary Table S1). Then, we synthesized single-site mutated primers (Supplementary Table S1) and constructed the expression vectors using pGEX-6p-1-eIF4A1 or pGEX-6p-1-eIF4A2 vectors as templates. PCR was performed in a final volume of 50 μL containing 25 μL 2X reaction buffer with 1.5 mM $MgCl_2$, 1.0 U PrimeStar polymerase, 2 μL forward and reverse primers (25 pmol each), 2 ng DNA template, and 23 μL distilled water. The PCR thermal cycling conditions were as follows: denaturation at 95 °C for 5 min, followed by 30 cycles at 95 °C for 30 s, 60 °C for 30 s, 72 °C for 1 min, and a final extension at 72 °C for 10 min. The PCR products were digested using the *Dpn*I enzyme to eliminate the template that produces the

original proteins. The digested products were transformed into *E. coli* DH5α competent cells, which were then grown on an LB plate with 100 mg/mL penicillin. The colonies were collected the next morning, inoculated in 5 mL LB broth containing 100 mg/mL penicillin, and cultured with shaking at 37 °C overnight. The plasmids were extracted using TIAN prep Mini Plasmid Kit II according to the manufacturer's protocol. The mutated vectors were validated by sequencing.

Expression and purification of mutated eIF4A1 and eIF4A2 proteins

The plasmids harboring mutated protein sequences were used to transform the *E. coli* BL21(DE3) strain. The positive colonies were further cultured at 37 ℃ with shaking overnight. The mutated proteins were expressed and purified using methods described for eIF4A1 or eIF4A2. The mutated proteins were concentrated and quantified using the Micro BCA Protein Assay Kit, and their purity was confirmed using PAGE. All mutated proteins had a molecular weight of approximately 70 kDa (**Supplementary Figure S6**).

Supplementary Table

Supplementary Table S1. Primers used in this study

Primer	Sequence
Primers used to amplify sequences encoding eIFA1 and eIF4A2 proteins	
6p-2-eIF4A1F	CGCGGATCCATGTCTGCGAGCCAGGATTC
6p-2-eIF4A1R	CCGCTCGAG TTATCAGATGAGG TCAGCAACAT
6p-2-eIF4A2F	CGCGGATCCATGTCTGGTGGCTCCGCGGATTATA
6p-2-eIF4A2R	CCGCTCGAGTTATTAAATAAGGTCAGCCACATTCATGG
Primers used to amplify sequences encoding mutated proteins	
4A1-Mut2-F	CAGCAGTTGGAGATTGAGTTCAAGGAGACCCAGGCCTTGGTCCTA
4A1-Mut2-R	CTGGGTCTCCTTGAACTCAATCTCCAACTGCTGCAGAATCGATAT
4A2-Mut2-F	CAGCAGATCGAGCTGGACCTGAAGGCCACCCAGGCCCTGGTGCTG GCC
4A2-Mut2-R	GGCCTGGGTGGCCTTCAGGTCCAGCTCGATCTGCTGCAGGATGCT
A1I94L-F	TCGATTCTGCAGCAGCTTGAATTAGATCTAAAA
A1I94L-R	TTTTAGATCTAATTCAAGCTGCTGCAGAATCGA
A1 L96I-F-2	ATTGAAATAGATCTAAAAGCCACCCAGGCCTTG
A1 L96I-R-2	TAGATCTATTTCAATCTGCTGCAGAATCGATAT
A1 D97E-F-2	GAATTAGAGCTAAAAGCCACCCAGGCCTTGGTC
A1 D97E-R-2	TTTTAGCTCTAATTCAATCTGCTGCAGAATCGA
A1 L98F-F-2	TTAGATTTTAAAGCCACCCAGGCCTTGGTCCTA
A1 L98F-R-2	TAGATCTATTTCAATCTGCTGCAGAATCGATAT
A1 D97E-F-2	GAATTAGAGCTAAAAGCCACCCAGGCCTTGGTC
A1 D97E-R-2	TTTTAGCTCTAATTCAATCTGCTGCAGAATCGA

A1 L98F-F-2	TTAGATTTTAAAGCCACCCAGGCCTTGGTCCTA
A1 L98F-R-2	GGCTTTAAAATCTAATTCAATCTGCTGCAGAAT
A1 A100E-F-2	CTAAAAGAGACCCAGGCCTTGGTCCTAGCACCC
A1 A100E-R-2	CTGGGTCTCTTTTAGATCTAATTCAATCTGCTG

Supplementary Figures

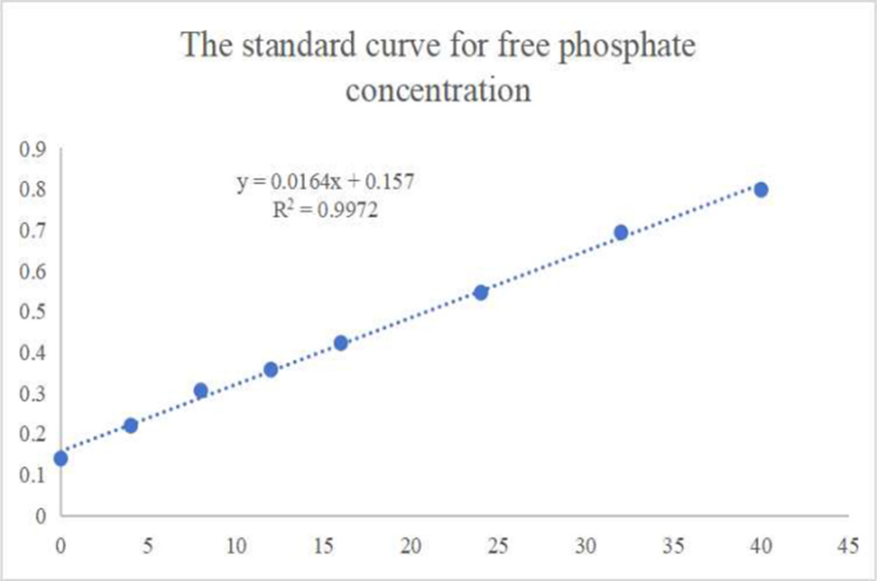

Supplementary Figure S1. The standard curve used to calculate the concentration of free phosphate. The absorbance at 620 nm of standard phosphate solutions (0, 4, 8, 12, 16, 24, 32, and 40 μM) were recorded and subjected to linear regression.

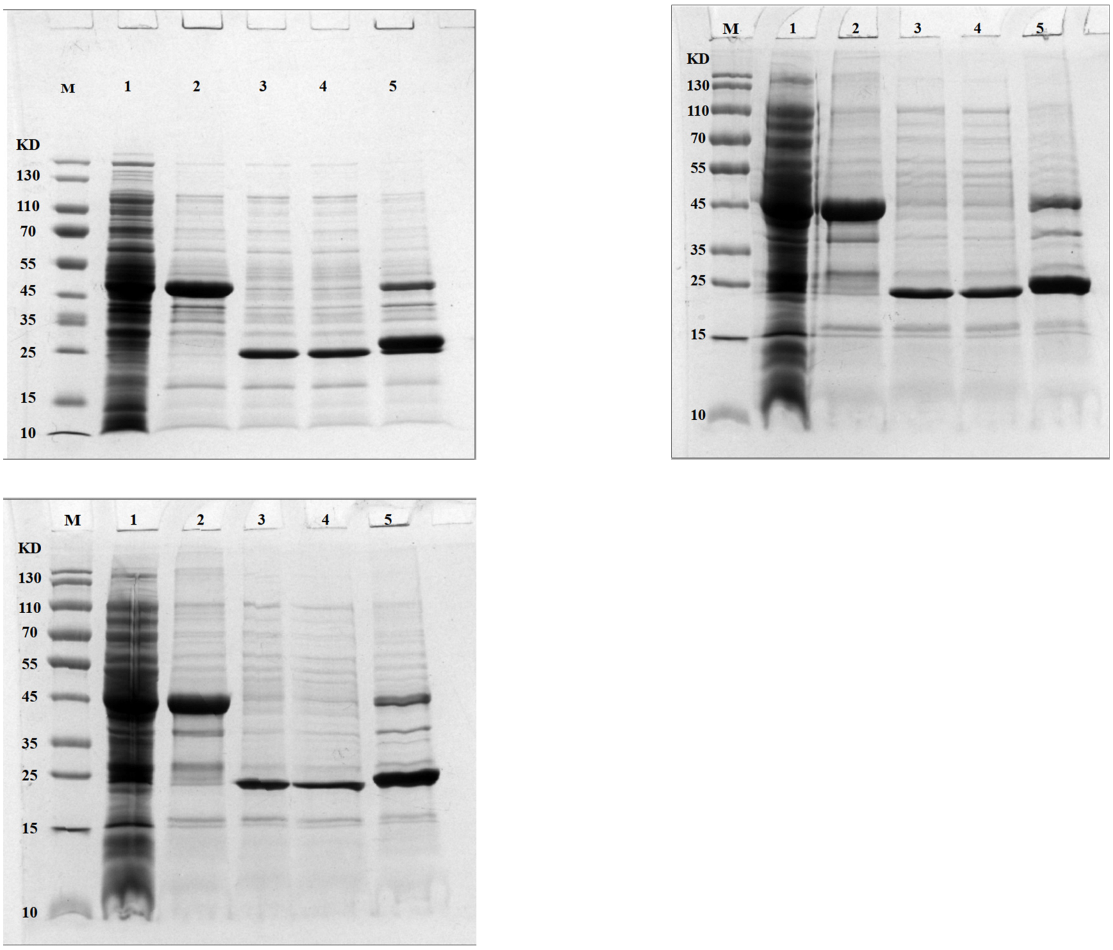

Supplementary Figure S2. BLF1 protein purification. M, standard protein marker; 1, supernatant of induced *E. coli* BL21(DE3) cells; 2, beads incubated with the supernatant; 3 and 4, PreScission Protease (PSP)-treated bead flow-out; 5, PSP-treated beads. Results from three independent purifications are shown to confirm experimental consistency.

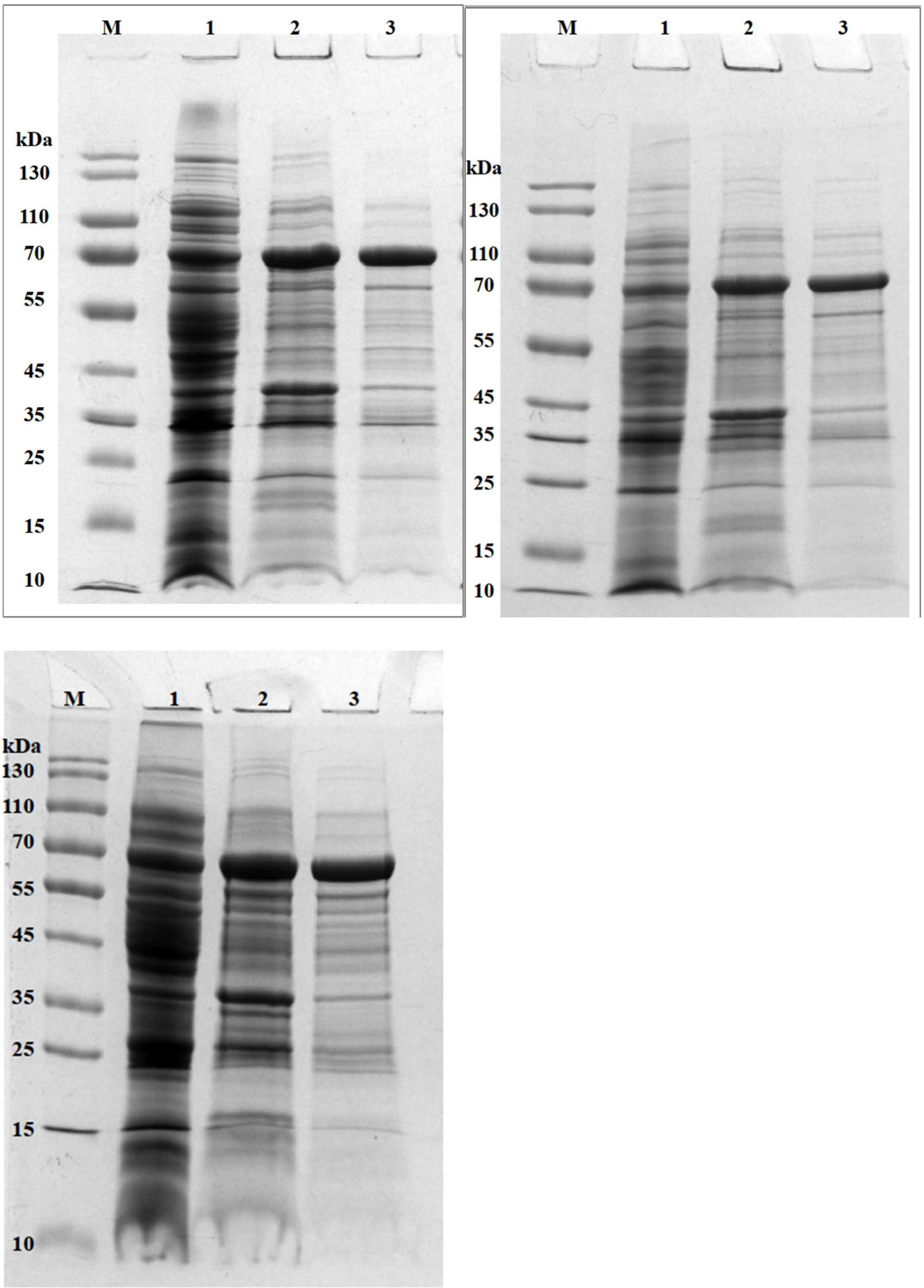

Supplementary Figure S3. eIF4A1 purification. M, standard protein marker; 1, supernatant of induced *E. coli* BL21(DE3) cells expressing eIF4A1; 2, beads incubated with cell supernatant; 3, Reduced glutathione (GSH)-treated bead flow-out. Results from three independent purifications are shown to confirm experimental consistency.

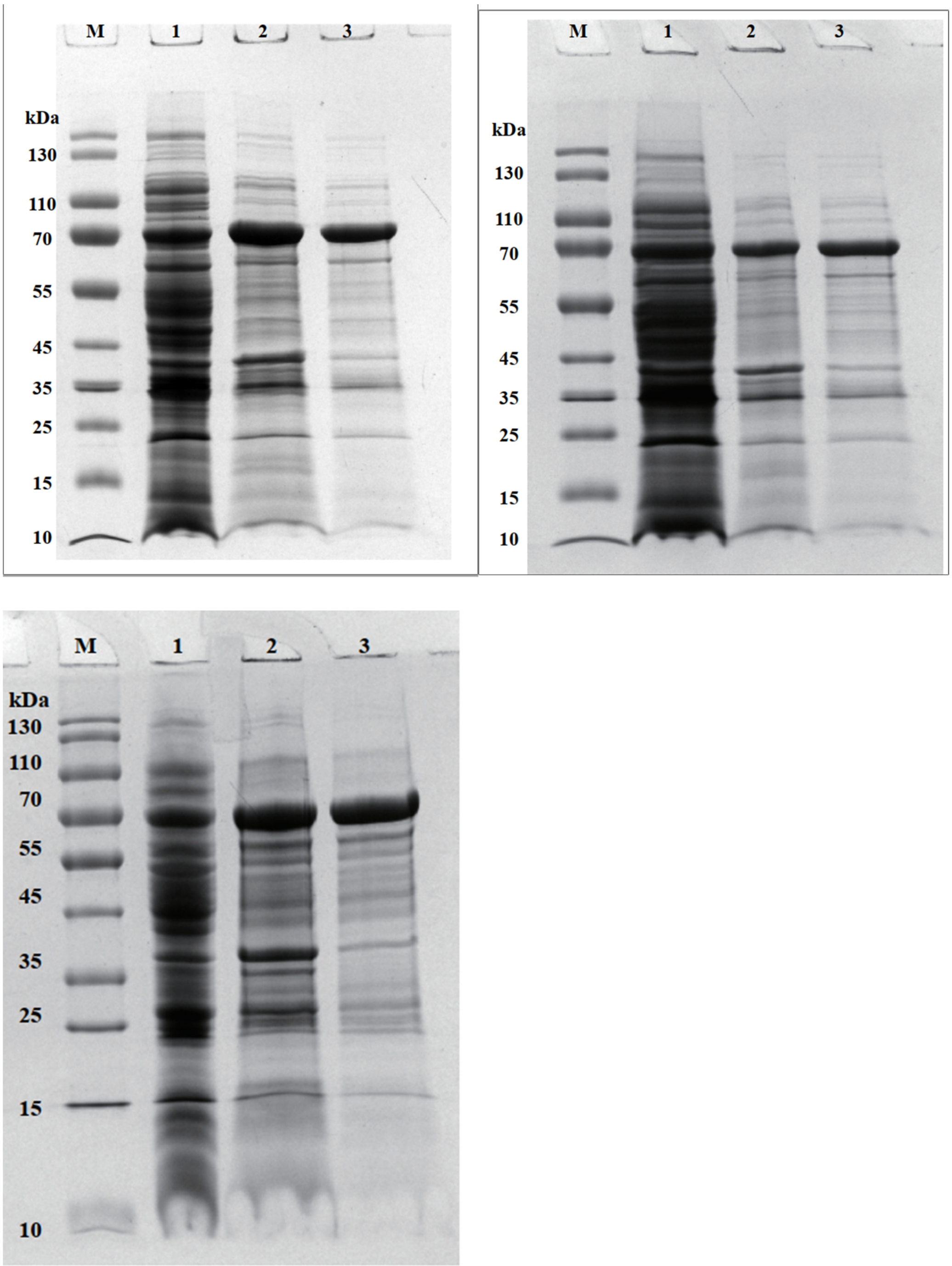

Supplementary Figure S4. eIF4A2 purification. M, standard protein marker; 1, supernatant of induced *E. coli* BL21(DE3) cells expressing eIF4A2; 2, beads incubated with cell supernatant; 3, GSH-treated bead flow-out. Results from three independent purifications are shown to confirm experimental consistency.

```
EIF4A1.SEQ   MSASQDSRSRDNG.PDGMEPEGVIESNWNEIVDSFDDMNL    39
EIF4A2.SEQ   MSGGSADYNREHGGPEGMDPDGVIESNWNEIVDNFDDMNL    40
Consensus    ms         r  g p gm p gviesnwneivd fddmnl

EIF4A1.SEQ   SESLLRGIYAYGFEKPSAIQQRAILPCIKGYDVIAQAQSG    79
EIF4A2.SEQ   KESLLRGIYAYGFEKPSAIQQRAIIPCIKGYDVIAQAQSG    80
Consensus     esllrgiyaygfekpsaiqqrai pcikgydviaqaqsg

EIF4A1.SEQ   TGKTATFAISILQQIELDLKATQALVLAPTRELAQQIQKV   119
EIF4A2.SEQ   TGKTATFAISILQQLEIEFKETQALVLAPTRELAQQIQKV   120
Consensus    tgktatfaisilqq e   k tqalvlaptrelaqqiqkv

EIF4A1.SEQ   VMALGDYMGASCHACIGGTNVRAEVQKLQMEAPHIIVGTP   159
EIF4A2.SEQ   ILALGDYMGATCHACIGGTNVRNEMQKLQAEAPHIVVGTP   160
Consensus      algdymga chaciggtnvr e qklq eaphi vgtp

EIF4A1.SEQ   GRVFDMLNRRYLSPKYIKMFVLDEADEMLSRGFKDQIYDI   199
EIF4A2.SEQ   GRVFDMLNRRYLSPKWIKMFVLDEADEMLSRGFKDQIYEI   200
Consensus    grvfdmlnrrylspk ikmfvldeademlsrgfkdqiy i

EIF4A1.SEQ   FQKLNSNTQVVLLSATMPSDVLEVTKKFMRDPIRILVKKE   239
EIF4A2.SEQ   FQKLNTSIQVVLLSATMPTDVLEVTKKFMRDPIRILVKKE   240
Consensus    fqkln   qvvllsatmp dvlevtkkfmrdpirilvkke

EIF4A1.SEQ   ELTLEGIRQFYINVEREEWKLDTLCDLYETLTITQAVIFI   279
EIF4A2.SEQ   ELTLEGIKQFYINVEREEWKLDTLCDLYETLTITQAVIFL   280
Consensus    eltlegi qfyinvereewkldtlcdlyetltitqavif

EIF4A1.SEQ   NTRRKVDWLTEKMHARDFTVSAMHGDMDQKERDVIMREFR   319
EIF4A2.SEQ   NTRRKVDWLTEKMHARDFTVSALHGDMDQKERDVIMREFR   320
Consensus    ntrrkvdwltekmhardftvsa hgdmdqkerdvimrefr

EIF4A1.SEQ   SGSSRVLITTDLLARGIDVQQVSLVINYDLPTNRENYIHR   359
EIF4A2.SEQ   SGSSRVLITTDLLARGIDVQQVSLVINYDLPTNRENYIHR   360
Consensus    sgssrvlittdllargidvqqvslvinydlptnrenyihr

EIF4A1.SEQ   IGRGGRFGRKGVAINMVTEEDKRTLRDIETFYNTSIEEMP   399
EIF4A2.SEQ   IGRGGRFGRKGVAINFVTEEDKRILRDIETFYNTTVEEMP   400
Consensus    igrggrfgrkgvain vteedkr lrdietfynt  eemp

EIF4A1.SEQ   LNVADL                                     405
EIF4A2.SEQ   MNVADL                                     406
Consensus     nvadl
```

Supplementary Figure S5. Blast results of eIF4A1 and eIF4A2 protein sequences.

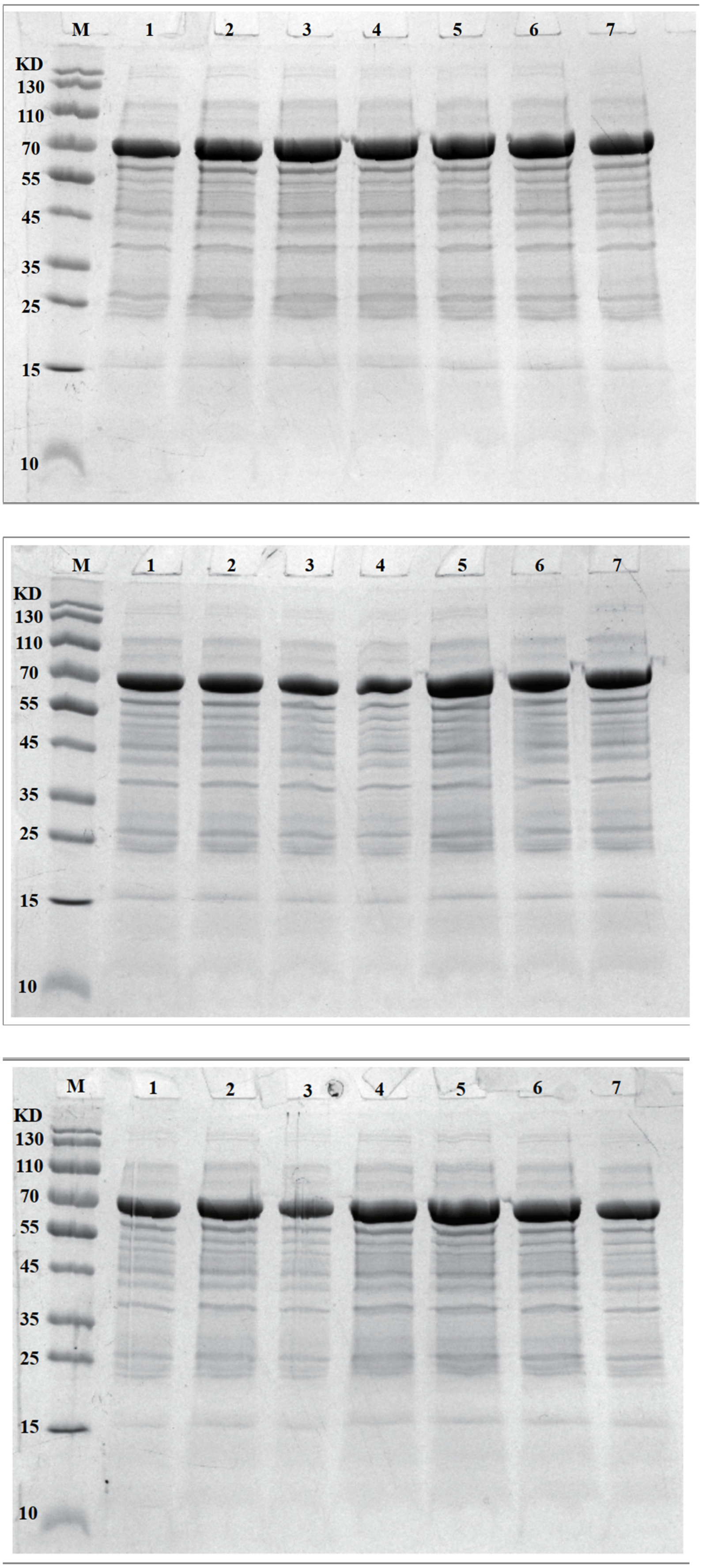

Supplementary Figure S6. Purified mutated proteins. 1, eIF4A1 Mut; 2, eIF4A2 Mut;

3, eIF4A1 I94L; 4, eIF4A1 L96I; 5, eIF4A1 D97E; 6, eIF4A1 L98F; 7, eIF4A1 A100E.

Three images represent three independent experiments.
